# A *MITF* Mutation Associated with a Dominant White Phenotype and Bilateral Deafness in German Fleckvieh Cattle

**DOI:** 10.1371/journal.pone.0028857

**Published:** 2011-12-12

**Authors:** Ute Philipp, Bettina Lupp, Stefanie Mömke, Veronika Stein, Andrea Tipold, Johanna Corinna Eule, Jürgen Rehage, Ottmar Distl

**Affiliations:** 1 Institute for Animal Breeding and Genetics, University of Veterinary Medicine Hannover, Hannover, Germany; 2 Clinic for Small Animals, University of Veterinary Medicine Hannover, Hannover, Germany; 3 Small Animal Clinic, Freie Universität Berlin, Berlin, Germany; 4 Clinic for Cattle, University of Veterinary Medicine Hannover, Hannover, Germany; University of Connecticut, USA, United States of America

## Abstract

A dominantly inherited syndrome associated with hypopigmentation, heterochromia irides, colobomatous eyes and bilateral hearing loss has been ascertained in Fleckvieh cattle (German White Fleckvieh syndrome). This syndrome has been mapped to bovine chromosome (BTA) 22 using a genome-wide association study with the bovine high density single nucleotide polymorphism array. An R210I missense mutation has been identified within *microphthalmia-associated transcription factor* (*MITF*) as responsible for this syndrome. The mutation is located in the highly conserved basic region of the protein and causes a negative-dominant effect. *SOX10* and *PAX3* promoter binding site mutations in *MITF* could be ruled out as causative for the German White Fleckvieh syndrome. Molecular characterization of this newly detected bovine syndrome means a large animal model is now available for the Tietz syndrome in humans.

## Introduction

A German Fleckvieh cattle family has been ascertained segregating for a white coat phenotype. Examination of these animals revealed a phenotype similar to incomplete albinism. Incomplete albinism in cattle is characterized by white coat color, pigmentless skin, heterochromia irides and white-yellowish hooves and horns [1], [2]. Incomplete albino cattle have previously been noted in Herefords and Hereford crossbreds. This condition has been considered as an autosomal dominant trait [3]. The German Fleckvieh investigated here distinguishes from Herefords and their crossbreds with incomplete albinism in that the German White Fleckvieh exhibited profound bilateral deafness. The German White Fleckvieh represents the first reported dominant white cattle with heterochromia irides and bilateral hearing loss. In dogs, white coat color and heterochromia irides are often associated with congenital sensorineural deafness [4]. Phenotypes similar to these dominant white cattle are known in humans as Waardenburg Syndrome Type 2A (WS2A) [5] and Tietz syndrome (TS) [6]–[8]. WS2A and TS are auditory-pigmentary syndromes caused by dominantly acting mutations in the *MITF* gene [9]. Patients with WS2A are experiencing sensorineural hearing loss of varying degrees and show heterochromia irides, early greying and a white forelock and in some cases vestibular dysfunction [5], [9]. Tietz syndrome differs in its phenotype from WS2A in some respects. Deafness is always congenital, profound and bilateral, depigmentation is generalized rather than patchy, but irides are not heterochromatic. Fundus hypopigmentation and vestibular dysfunction are similar in both syndromes, WS2A and TS [6]–[9]. The objectives of this study were to characterize the phenotypes of German White Fleckvieh and to identify the mutation responsible for this newly detected phenotype in cattle using genome-wide association analyses and re-sequencing of *MITF*, the most likely candidate gene.

## Results

### Phenotypic characterization of the animals

**Figure 1:**
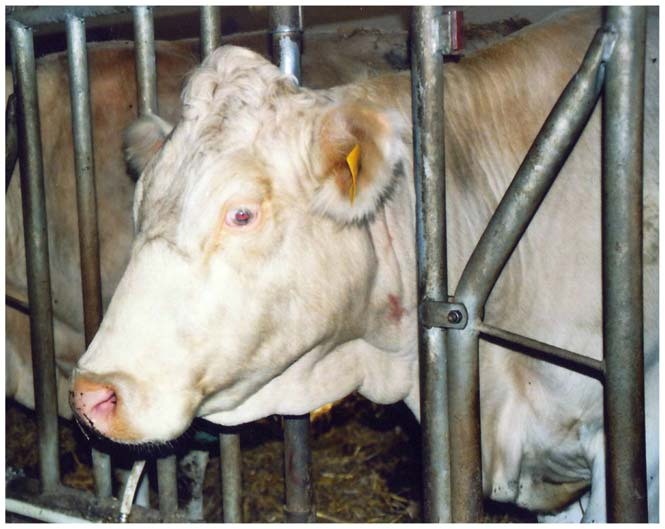
German White Fleckvieh cow exhibiting a colour mutation similar to incomplete albinism with irises being pale blue in the central part and white towards the periphery as well as pigmentless muzzle, eyelashes and eyelids.

**Figure 2:**
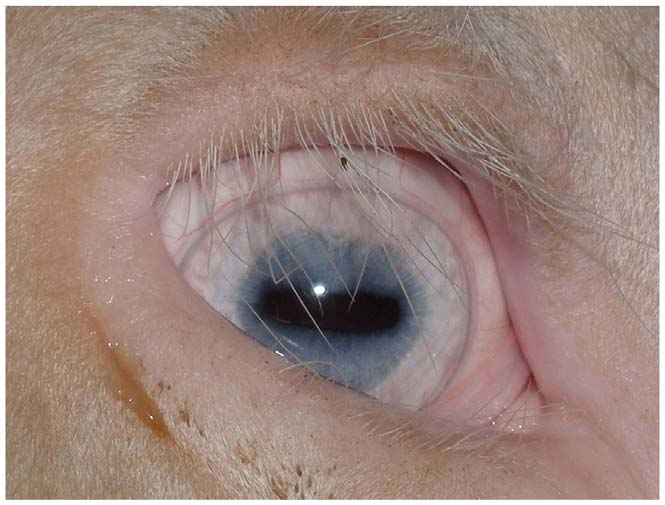
Heterochromia irides with the central iris appearing blue and white towards the periphery in the right eye of a German White Fleckvieh bull. Cilia, eyelashes, eyelids, nictitating membrane and conjunctiva are white.

**Figure 3:**
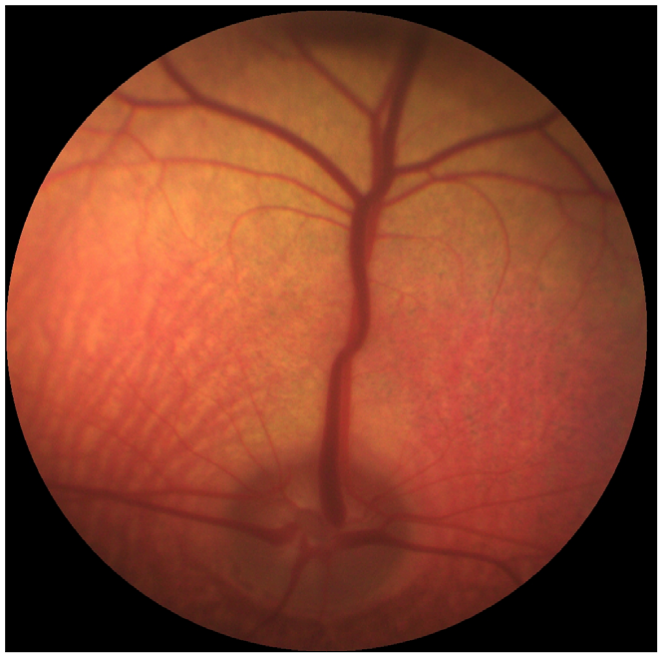
Ocular fundus of the left eye of a German White Fleckvieh bull showing a subalbinotic fundus with large areas of choriodal hypopigmentation. Choriodal vessels and white sclera are visible due to the transparent choriodal layer.

**Figure 4:**
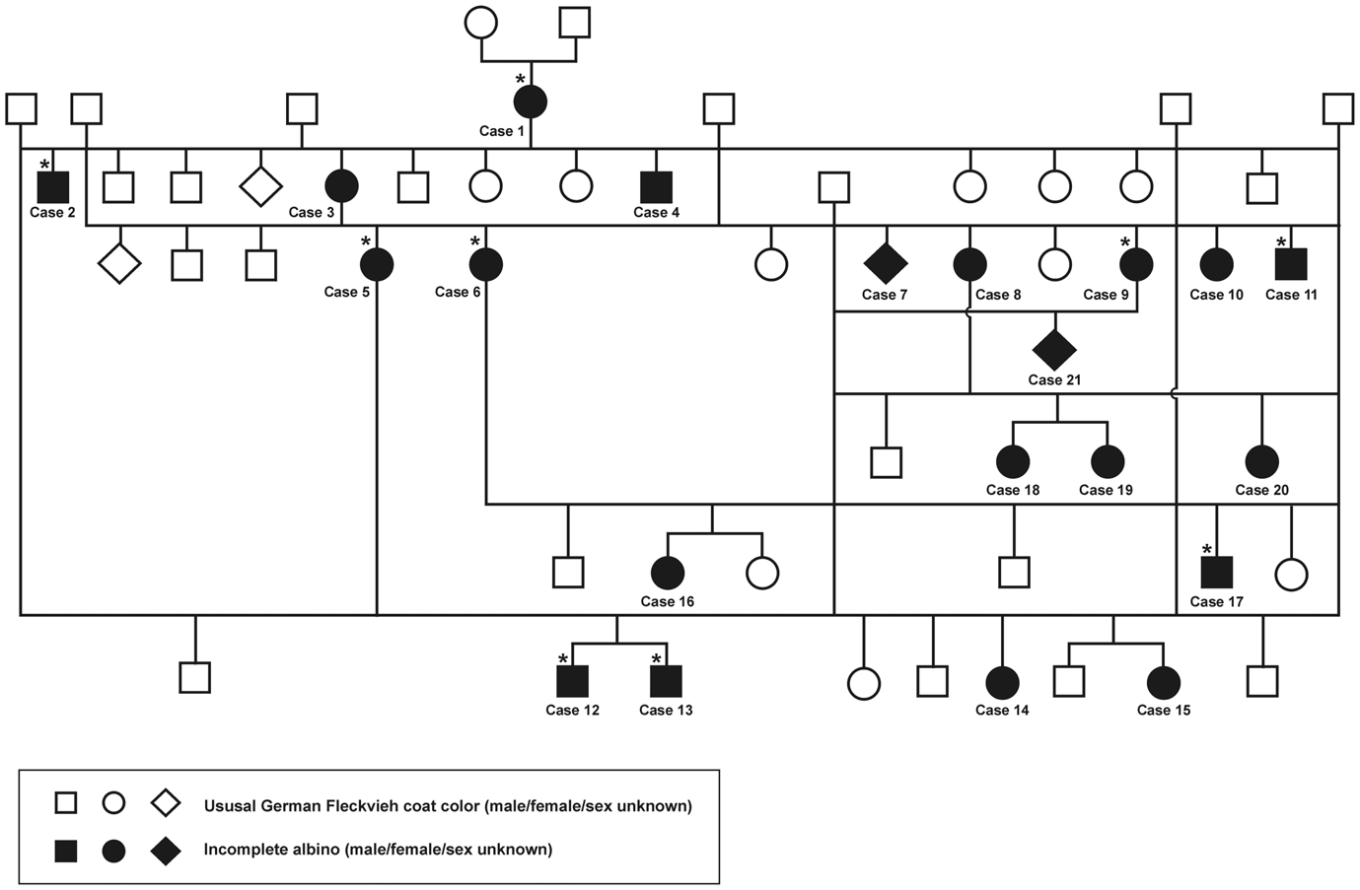
Pedigree of the German White Fleckvieh family resembling an incomplete albino phenotype. The founder animal for all white animals is Case 1. All animals marked with a star were examined on the farm and genotyped for the c.629G>T and g.32387485A>G mutations. Not all spotted progeny are depicted in the pedigree. With exception of Case 13 and Case 17, all white animals marked with a star were genotyped using the *bovine* high density Illumina beadchip.

The German White Fleckvieh cattle family exhibited pure white coat color, pink skin without any darker spots or ghost patterns, yellow-white hooves, white horns, pigmentless muzzle, anus, eyelids, eye lashes, cilia, nictitating membranes and conjunctivae (Figure 1). The irides were pale blue in the central part and white towards the periphery (Figure 2). Pupils exhibited typical albinotic light reflection and the ocular fundus was completely or nearly completely albinotic using direct ophthalmoscopy (Figure 3). Optic disks were enlarged and irregular. The white animals were bilaterally deaf. We could not observe behavioural reactions or Preyer's reflex on different sounds. Sensorineural hearing loss could be confirmed using brainstem auditory evoked response (BAER) tests in two young bulls. The mode of inheritance appeared autosomal dominant with full penetrance and all white cattle could be traced to the same white founder dam (Figure 4).

### Mapping the associated genomic region

A genome-wide association study (GWAS) using the *bovine* high density Illumina bead chip with 774,660 single nucleotide polymorphisms (SNPs) and TASSEL [10], [11] revealed the genome-wide most significantly (−log_10_P_raw_ = 254.4; −log_10_P_Bonferroni-adjusted_ = 248.6) associated region on bovine chromosome (BTA) 22 at 33.422, 36.052 and 36.060 Mb (Figure S1). Haplotypes were estimated using these three SNPs and the procedure HAPLOTYPE of SAS/Genetics, version 9.2. The haplotypes of dominant white animals were consistent with inheritance of the mutation from the common founder dam and one joint haplotype for the alleles associated with the dominant white mutation could be confirmed (Table S1). The alleles of the associated haplotype were uncommon in German Fleckvieh and not present in the spotted animals related with German White Fleckvieh.

### Identification of the associated mutation

**Figure 5:**
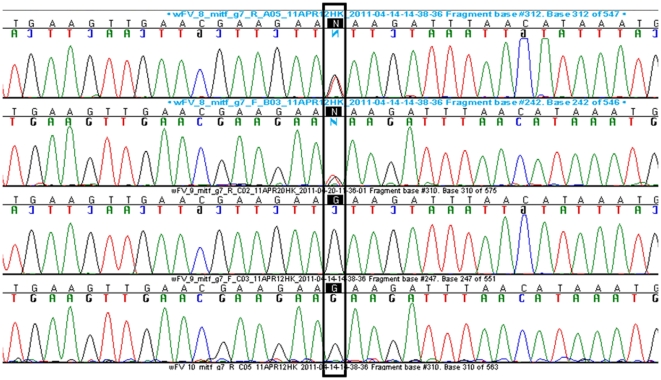
Sequences of exon 7 of the *MITF* M-form of a dominant German White Fleckvieh and a spotted German Fleckvieh animal. Base pairs in boxes demonstrate the c.629G>T mutation causing the Tietz-like-syndrome in German White Fleckvieh. The first row shows the forward and the second row the reverse sequence of an affected animal with the heterozygous G/T genotype, the third and fourth row contain the forward and the reverse sequence of a spotted German Fleckvieh animal with the homozygous G/G wildtype.

Among 13 genes contained in the associated interval on BTA22 of Btau 4.0/5.2 (Ensemble, http://www.ensembl.org/index.html), we considered the *microphthalmia-associated transcription factor* (*MITF*) gene at 32,353,746–32,387,234 bp as the most plausible candidate. MITF is essential for the development and post-natal survival of melanocytes. All *MITF* mutations reported in humans and mice affect melanocytes to a varying degree, retinal pigment epithelial cells and a few osteoclasts. The resulting phenotypic changes are characterized by pigmentary defects of skin, coat and eyes, deafness and in some instances osteopetrosis or hyperosteosis [9]. We could not see genome-wide significant signals in the GWAS at BTA2, 5, 6, 12, 15, and 29 where possible candidate genes other than *MITF* are located on the bovine genome assembly. Thus, possible candidates for white coat colour (*KIT* on BTA6, *KITLG* on BTA5, *PMEL* on BTA5, *TYR* on BTA29) and other Waardenburg syndromes (WS) including *PAX3* (WS1, WS3; on BTA2), *EDNRB* (WS4a; on BTA12), *EDN3* (WS4b; on BTA13) and *SOX10* (WS2e, WS4c; on BTA5) could be clearly excluded. MITF is a transcription factor of the basic helix-loop-helix zipper (bHLH-Zip) family of proteins controlling differentiating and development of melanocytes, osteoclasts and mast cells [12]. We sequenced all exons of all *in silico* identified bovine *MITF* isoforms (Table S2, Figure S2). A missense mutation (c.629G>T, p.210R>I) was identified within exon 7 of the bovine *MITF* M-Form (NM_001001150) for which all German White Fleckvieh animals were heterozygous (Figure 5). Furthermore, this mutation was not detected in 383 non-completely white controls from ten different breeds (Table S3). *In silico* analysis using Polyphen predicted a probably damaging effect on the protein with a PSIC score difference of 3.065. The c.629G>T mutation is localized within the region encoding the basic region of the MITF protein involved in DNA binding. The basic domain is highly conserved between all species including vertebrates and invertebrates (Table 1) [9]. In order to evaluate possible effects on *MITF* expression through mutations in the binding sites of *SOX10* and *PAX3*, we analyzed the *MITF* M-form promoter region for all previously reported binding sites of *SOX10* and *PAX3* [9], [13]. Both genes, *SOX10* and *PAX3* act directly and synergistically to activate the *MITF* promoter. Mutations in the binding sites of the *MITF* promoter can abolish transcriptional activation of *MITF* [13], [14]. We detected eight putative *SOX10* and two putative *PAX3* binding sites after screening approximately 1 kb of the bovine reference sequence Btau 5.2 upstream of the 5′end of *MITF* transcription start site. Therefore, we sequenced a 581 bp amplicon containing the putative eight *SOX10* and the two putative *PAX3* binding sites. Mutation screening revealed a g.32387485A>G SNP at position −212 of the putative *SOX10* binding site 7/8. However, this binding site showed only minor effects on transcriptional activity in co-transfection assays with HeLa cells [13]. The mutant G allele appeared generally common in Fleckvieh and even more frequently distributed among German White Fleckvieh. Eight of the nine German White Fleckvieh cattle and two of the spotted German Fleckvieh cattle were homozygous for the G allele (Table 2). At the putative *PAX3* binding sites, we could not detect any mutations.

**Table 1:** Protein sequence alignment of the MITF basic helix-loop-helix (bHLH) domain in vertebrate species.

Species/Breed	Protein identifier	Protein sequence
German White Fleckvieh		SEARALAKERQKKDNHNLIERR***I***RFNINDRIKELGTLIPKSNDPDMRWNKGTILKASV
Cow	ENSBTAP00000053693	SEARALAKERQKKDNHNLIERR***R***FNINDRIKELGTLIPKSNDPDMRWNKGTILKASV
Human	ENSP00000295600	********************** R **********************************
Mouse	ENSMUSP00000044938	********************** R **********************************
Rat	ENSRNOP00000044350	********************** R **********************************
Dog	ENSCAFP00000009730	********************** R **********************************
Horse	ENSECAP00000004487	********************** R **********************************
Dolphin	ENSTTRP00000006690	********************** R **********************************
Elefant	ENSLAFP00000017388	********************** R **********************************
Kangaroo rat	ENSDORP00000003119	********************** R **********************************
Chicken	ENSGALP00000012434	********************** R **********************************
Frog	ENSXETP00000000313	********************** R **********************************
Lion's cove	BAD69632	A*V******************* R **********************************

**Table 2:** Genotype and allele frequencies in German White Fleckvieh and German Fleckvieh genotyped for the g.32387485A>G *MITF* promoter mutation.

Breed	n	Coat color	Number of animals			Allele frequency
			G/G	G/A	A/A	G
German White Fleckvieh	′	white	8	1	-	0.947
German Fleckvieh related with German White Fleckvieh	6	spotted	2	3	1	0.583

## Discussion

In German White Fleckvieh, a perfect cosegregation was demonstrated for a missense mutation affecting the DNA binding domain of *MITF*. Function of protein domains was shown in detail for transcription factor TFEB [15] which shares critically conserved residues with MITF and other bHLH-Zip proteins [16], [17]. Arginine210 seems to be necessary to contact the phosphate backbone of DNA and to build a hydrogen bond to an N-terminal glutamine. In German White Fleckvieh, the p.210R>I substitution probably disrupts both functions as the strong basic amino acid arginine is replaced by the non-polare isoleucine. In humans, the Tietz syndrome is characterized by a missense mutation (N210K) [18] or a 3-bp in-frame deletion (DelR217) [7], [8]. In the semi-dominant oak ridge mouse mutant (*Mitf^mi-or^*), the corresponding arginine is replaced by lysine (R216K) which affects the DNA binding domain [19]. In mice, further semi-dominant mutations affecting the DNA binding domain of MITF were seen in the *Mitf^mi^*, *Mitf^mi-wh^*, *Mitf^mi-b^*, *Mitf^mi-H^*, *Mitf^mi-enu5^*, *Mitf^mi-bcc2^* and *Mitf^mi-ew^* mutations [9]. The *Mitf^mi^* mutation in the mouse is identical to the human DelR217 mutation. It is worth to mention that the bovine R210I and the human DelR217 and N210K mutations lead to more similar phenotypes than the murine mutations in this critical region. In humans and cattle, the mutations cause a dominant-negative effect with no variable expressivity. Humans and cattle, both are born white and sensorineural hearing loss was always bilateral [6]–[9], [18]. In contrast to humans, cattle show heterochromia irides and this could be caused by the substitution of the non-polare isoleucine amino acid. In mice however, the trait is transmitted in a semi-dominant or recessive fashion and phenotypes differ from human WS2A and TS [9] as well as the German White Fleckvieh reported here. In heterozygous mice, white spotting and/or color dilution is observed and in some cases reduced iris pigment. In the homozygous condition, microphthalmia or even absent eyes, osteopetrosis and in some inner ear defects and reduced fertility have been noted [9]. In humans with WS2A or TS and in addition, in German White Fleckvieh, neither eye defects nor osteopetrosis have been found.

The homozygous genotype of the mutation of the putative *SOX10* binding site 7/8 was not specific for the German White Fleckvieh and thus, an effect on the white phenotype can be ruled out. However, this *MITF* promoter mutation could be associated with the distribution of the white coat color in spotted German Fleckvieh because the bovine reference sequence was from an animal of the Hereford breed.

In conclusion, we have identified the mutation responsible for a Tietz-like-syndrome in cattle. This is the first time that an unambiguous mutation in the *MITF* gene is identified in non-human and non-rodent mammals. The German White Fleckvieh is a useful large animal model to study human Tietz syndrome and the MITF transcription factor network. This study highlights the strength of trait mapping using high density SNP arrays in domestic animals, particularly with regard to one or a few founder animals for the target trait.

## Materials and Methods

### Ethics statement

All animal work has been conducted according to the national and international guidelines for animal welfare. The study has been approved by the Lower Saxony state veterinary office Niedersächsisches Landesamt für Verbraucherschutz und Lebensmittelsicherheit, Oldenburg, Germany, and registered under the approval ID 33.42502-05-04A247.

### Animals

The genome-wide association study (GWAS) included genomic DNA samples of seven white and 79 spotted German Fleckvieh whereof five samples of the spotted German Fleckvieh were from the same herd as the German White Fleckvieh. From the seven white animals, three animals were males and four females. In controls, females and males were at the same proportions. DNA samples of nine German White Fleckvieh, six German Fleckvieh related with German White Fleckvieh, 89 German Fleckvieh unrelated with German White Fleckvieh and 288 animals from nine other breeds and German Fleckvieh x Red Holstein crossbred animals were used to test the *MITF* c.629G>T mutation. For cDNA analysis, hair root samples of two white males and one spotted female were used.

On the farm where the German White Fleckvieh had been detected, only the female white animals were used for breeding, whereas all bull calves were used for fattening without siring offspring. Therefore, all white animals had a white mother and a normally spotted German Fleckvieh sire (Figure 4). In total, six female colour variants gave birth to 20 white animals and 24 spotted offspring. Matings among spotted descendants with German Fleckvieh sires did not result in white offspring. The ratio of white to spotted offspring from the six white dams used for breeding was close to the 1∶1 ratio expected for a monogenic autosomal dominant mode of inheritance. All other modes of inheritance are unlikely. The colour variant of the founder cow appears to be caused by a spontaneous mutation as both parents were normally spotted and other white offspring from the parents of the founder dam had not been reported.

### Genotyping and genome-wide association analysis

DNA from seven dominant white German Fleckvieh (cases) and 79 spotted German Fleckvieh cattle (controls) randomly sampled from the whole German Fleckvieh cattle population were genotyped using the *bovine* Illumina high density beadchip with 774,660 single nucleotide polymorphisms (SNPs). Quality control for genotyping was performed using two duplicates. Consistency of genotyping was >0.999. After filtering for a minor allele frequency >0.05 and genotyping rate >0.99, 585,047 SNPs were left for a genome-wide association study (GWAS) among cases and controls. GWAS was performed using Tassel, version 3.0 (URL:http://www.maizegenetics.net/index.php?option=com_content&task=view&id=89&Itemid=119). A general model was employed to control for the data structure in GWAS. The fixed effects in the model included sex and the first eight principal components. Principal components were estimated using TASSEL and a pruned set of SNPs. A sliding window size of 50 SNPs, window shift steps of five SNPs and a pairwise correlation (r^2^)<0.2 were used for generating this set of 56,535 SNPs. In order to test the consistency of the model, the first four, first six or first ten principal components were employed in additional models. The association results were consistent among the different models and therefore, data from these models are not shown. Genome-wide significance was ascertained through a Bonferroni correction for the number of SNPs tested. Association of haplotypes with the dominant white phenotype was tested using the procedure HAPLOTYPE of SAS/Genetics (Statistical Analysis System, version 9.2, Cary, NC, USA, 2011, http://support.sas.com/documentation /cdl_main/index.html).

### DNA sequence analysis

Genomic DNA was isolated from frozen EDTA stabilized blood samples using an in-house desalting method. For identifying DNA polymorphisms within the bovine *MITF* gene (Gene ID: 407219), the putative exons of the *MITF* gene were determined by comparative alignment of the bovine genomic sequence ((NC_007320.4, 157513 bp–618513 bp) versus the known bovine mRNA isoform M (NM_001001150) and the eight human *MITF* mRNA isoform sequences (NM_000248.3, NM_001184967.1, NM_001184968.1, NM_006722.2, NM_198158.1, NM_00198159.1, NM_00198177.1 and NM_00198178.1) using Spidey (http://www.ncbi.nlm.nih.gov/blast/). For sequence analysis, primer pairs for all identified exons and adjacent intronic regions were designed using PRIMER3 software (http://frodo.wi.mit.edu/cgi-bin/primer3/primer3_www.cgi).

For mutation analysis of the *MITF* M-form promoter the previously reported murine *SOX10* and *PAX3* binding sites were identified by searching their core sequences [9] in the bovine reference sequence Btau 5.2 upstream of the 5′end of the putative *MITF* transcription site. Primer pairs were designed for a 581 bp amplicon that contained all previously reported binding sites for *SOX10* (sites S1–S8) and *PAX3* (sites P1–P2) [9].

PCR was carried out in 30 µl containing 10 ng genomic DNA according to the standard protocol advised by the manufacturer of the *Taq* DNA polymerase (Qbiogene Heidelberg, Germany). The subsequent sequencing of the PCR products was performed using the ABI BigDye Terminator v3.1 sequencing kit (Life Technologies, Darmstadt, Germany). The products were analyzed on an automated ABI 3500 capillary sequencer (Life Technologies).

### cDNA sequence analysis

RNA was extracted from hair root cells using the RNeasy Universal Tissue kit (Qiagen, Hilden, Germany). The following cDNA synthesis was carried out in a 20 µl reaction volumes using Maxima First strand cDNA synthesis kit (Fermentas, St. Leon-Rot, Germany). The open reading frame of *MITF* was generated with two amplicons. In addition, specific primer pairs were designed to amplify the predicted different *MITF* isoforms (Table S2). The RT amplicons were directly sequenced using the PCR primer pairs for sequencing. Sequencing was performed using the BigDye Terminator v3.1 sequencing kit (Life Technologies). The products were analyzed on an ABI 3500 capillary sequencer (Life Technologies).

### SNP detection using the ABI SNP detection system

Primer pairs and probes for the *MITF* c.629G>T SNP were designed using the Custom Taqman SNP genotyping assay service (Table S2). MGB probes were dye-labelled with FAM and VIC, respectively. Genotyping was performed in 12.0 µl reaction volumes containing 6.0 µl SensiMix DNA Kit (Quantace, London, UK), 0.3125 µl 40×Assay (Applied Biosystems, Darmstadt, Germany) and 1.5 µl genomic DNA on an ABI 7300 sequence detection system (Life Technologies). Reaction conditions for a two step PCR were set using ABI instructions. Test samples were run in single reactions, controls for homozygous T/T and G/T genotypes were run in duplex on every analyzed plate. DNA samples of dominant white cattle were used as heterozygous controls while the homozygous T/T genotypes were generated by cloning a heterozygous Mitf_Ex7_M-form amplicon using the Qiagen PCR cloning kit (Qiagen). Clones containing the strand with the mutated base T were confirmed by sequencing.

All new sequence data and polymorphisms from this study have been submitted to the EMBL/GenBank Data Libraries.

## Supporting Information

Figure S1 **Manhattan plot of the −log_10_ P-values for the genome-wide association analysis of the dominant white phenotype in German Fleckvieh from a general model analysis using TASSEL, version 3.0.** The highest −log_10_ p-values (254) were obtained for bovine chromosome 22 at 33 and 36 Mb.
(DOC)

Figure S2 ***In silico*** **identified bovine** ***MITF*** **isoforms using bovine genomic** ***MITF*** **gene sequences (Gene ID: 407219), bovine mRNA sequences of the** ***MITF*** **isoform M (NM_001001150) and all eight human** ***MITF*** **mRNA isoform sequences (NM_000248.3, NM_001184967.1, NM_001184968.1, NM_006722.2, NM_198158.1, NM_00198159.1, NM_00198177.1 and NM_00198178.1).**
(DOC)

Table S1 **Haplotype frequencies and their standard errors (SE) for all animals, German White Fleckvieh (GWF) and controls and haplotype-trait association with χ^2^- and P-values.** The founder dam haplotye conforms to G-A-A.
(DOC)

Table S2 **Primer pairs used for sequencing the bovine** ***MITF*** **gene and mutation detection, amplicon size and annealing temperature (AT) for the amplicons.**
(DOC)

Table S3 **Number of animals (n) per breed, their coat color and genotype for the c.629G>T** ***MITF*** **mutation.**
(DOC)
