# Supplementary material for: A MITF Mutation Associated with a Dominant White Phenotype and Bilateral Deafness in German Fleckvieh Cattle
Source: PLoS One. 2011 Dec 12;6(12):e28857. doi: 10.1371/journal.pone.0028857 (PMC3236222; doi:10.1371/journal.pone.0028857)
Supplement: Figure S1 — Manhattan plot of the −log10 P-values for the genome-wide association analysis of the dominant white phenotype in German Fleckvieh from a general model analysis using TASSEL, version 3.0. The highest −log10 p-values (254) were obtained for bovine chromosome 22 at 33 and 36 Mb. (DOC) [file pone.0028857.s001.doc]

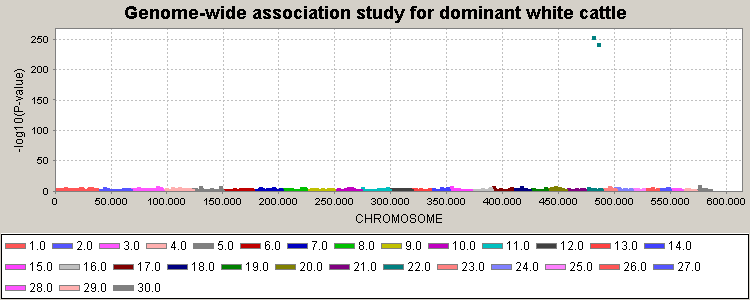


**Figure S1.** Manhattan plot of the –log_10_ P-values for the genome-wide association analysis of the dominant white phenotype in German Fleckvieh from a general model analysis using TASSEL, version 3.0. The highest –log_10_ p-values (254) were obtained for bovine chromosome 22 at 33 and 36 Mb.
