# Supplementary material for: A MITF Mutation Associated with a Dominant White Phenotype and Bilateral Deafness in German Fleckvieh Cattle
Source: PLoS One. 2011 Dec 12;6(12):e28857. doi: 10.1371/journal.pone.0028857 (PMC3236222; doi:10.1371/journal.pone.0028857)
Supplement: Figure S2 — In silico identified bovine MITF isoforms using bovine genomic MITF gene sequences (Gene ID: 407219), bovine mRNA sequences of the MITF isoform M (NM_001001150) and all eight human MITF mRNA isoform sequences (NM_000248.3, NM_001184967.1, NM_001184968.1, NM_006722.2, NM_198158.1, NM_00198159.1, NM_00198177.1 and NM_00198178.1). (DOC) [file pone.0028857.s002.doc]

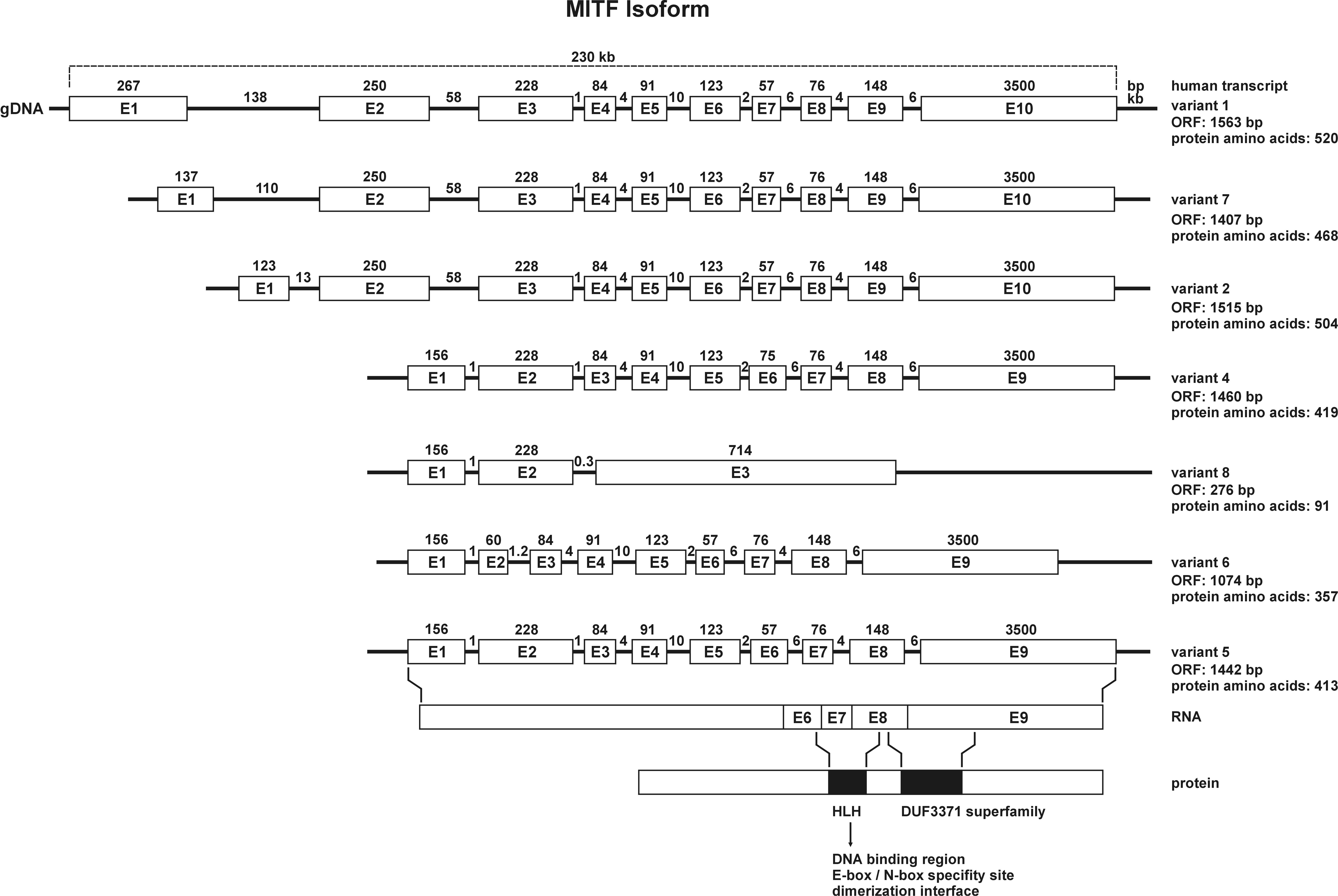


**Figure S2.** *In silico* identified bovine *MITF* isoforms using bovine genomic *MITF* gene sequences (Gene ID: 407219), bovine mRNA sequences of the *MITF* isoform M (NM_001001150) and all eight human *MITF* mRNA isoform sequences (NM_000248.3, NM_001184967.1, NM_001184968.1, NM_006722.2, NM_198158.1, NM_00198159.1, NM_00198177.1 and NM_00198178.1). Exons are numbered as E1 to E10, numbers above the boxes give the number of base pairs for the respective exon, lines between exons indicate introns, their size is given in base pairs above the line. For each isoform the size of the open reading frame (ORF) in base pairs (bp) and the number of amino acids are given. The helix-loop-helix domain (HLH) and DUF3371 superfamily is represented by a black box for the MITF protein and their encoding exonic regions.
