# Supplementary material for: A MITF Mutation Associated with a Dominant White Phenotype and Bilateral Deafness in German Fleckvieh Cattle
Source: PLoS One. 2011 Dec 12;6(12):e28857. doi: 10.1371/journal.pone.0028857 (PMC3236222; doi:10.1371/journal.pone.0028857)
Supplement: Table S1 — Haplotype frequencies and their standard errors (SE) for all animals, German White Fleckvieh (GWF) and controls and haplotype-trait association with χ2- and P-values. The founder dam haplotye conforms to G-A-A. (DOC) [file pone.0028857.s003.doc]

**Table S1.** Haplotype frequencies and their standard errors (SE) for all animals, German White Fleckvieh (GWF) and controls and haplotype-trait association with χ^2^- and P-values. The founder dam haplotye conforms to G-A-A.

| Haplotype | Frequency | SE | Frequency | | χ^2^-value | P-value |
| --- | --- | --- | --- | --- | --- | --- |
|  |  |  | Controls | GWF |  |  |
| A-C-G | 0.862 | 0.026 | 0.903 | 0.357 | 32.32 | <0.0001 |
| G-C-G | 0.072 | 0.019 | 0.074 | 0.071 | 0.001 | 0.973 |
| G-A-A | 0.049 | 0.016 | 0.000 | 0.571 | 90.08 | <0.0001 |
| A-A-A | 0.017 | 0.010 | 0.022 | 0.000 | 0.288 | 0.592 |

Haplotypes include the SNPs BovineHD2200009335, BovineHD2200010093 and BovineHD2200010095 at 33.422, 36.052 and 36.060 Mb on BTA22.
