# Supplementary material for: A MITF Mutation Associated with a Dominant White Phenotype and Bilateral Deafness in German Fleckvieh Cattle
Source: PLoS One. 2011 Dec 12;6(12):e28857. doi: 10.1371/journal.pone.0028857 (PMC3236222; doi:10.1371/journal.pone.0028857)
Supplement: Table S2 — Primer pairs used for sequencing the bovine MITF gene and mutation detection, amplicon size and annealing temperature (AT) for the amplicons. (DOC) [file pone.0028857.s004.doc]

**Table S2.** Primer pairs used for sequencing the bovine *MITF* gene and mutation detection, amplicon size and annealing temperature (AT) for the amplicons.

| Amplicon name | Target | Forward primer (5’→3’) | Reverse primer (5’→3’) | Amplicon size | AT [°C] |
| --- | --- | --- | --- | --- | --- |
| Mitf- M promoter | gDNA | GATGCAAGATGAGACTGCTG | CCCAATCCAACAAGAAACAG | 581 bp | 57 |
| Mitf_Ex1_iso-1 | gDNA | TGAATCCAAACAGGAGTTGC | CTTTCCTCGCCTCAGAGTG | 525 bp | 58 |
| Mitf_Ex1_iso-2 | gDNA | CTTGTGAAGCCCCTGTTG | ACAGCTGAAGCCGATTAAAG | 464 bp | 57 |
| Mitf_Ex1_iso-4 | gDNA | AGCAAACTTGTAGGGCTTCC | TGGATTACTGCTTATGGCATC | 431 bp | 57 |
| Mitf_Ex1_iso-7 | gDNA | GATTTGAATGAGAGCCAAGC | TTCACAGTGTCCCATCTGAG | 485 bp | 57 |
| Mitf_Ex2_M-form | gDNA | CCAAATCCGAAGAGTTAGCC | GGAGCCATAACCCAATAACC | 620 bp | 58 |
| Mitf_Ex2_iso1-2-3-7 | gDNA | TCCTGTCAGCTCTTGATTTTC | AGCCATGTAGGAATCAATGG | 569 bp | 57 |
| Mitf_Ex3_M-form | gDNA | CTGTCCATGCTTGTCATCTG | CCAAAGTCATTAGGAAGGTGTG | 365 bp | 57 |
| Mitf_Ex3_isoform-8 | gDNA | TGTTCTCTTCCATGCCTTTC | CTGCCGAGTCATTCAGTACC | 851 bp | 58 |
| Mitf_Ex4_M-form | gDNA | TCCTCCTCATCACCCATTAG | GACTCCAGGGAATGCAGAG | 521 bp | 57 |
| Mitf_Ex5_M-form | gDNA | TCTGGAATAGGATAAGGGTTTTAG | AGACTGGGAGTGTTCTGCTC | 495 bp | 57 |
| Mitf_Ex6_M-form | gDNA | GTTAAGTCAGATAAACATCCATTTG | GGGATCAAAATTCCCTCTTAC | 455 bp | 56 |
| Mitf_Ex7_M-form | gDNA | TGGTAGCTCCAATTTCAAGAG | ACCTTCCCACCTCCAAAG | 593 bp | 57 |
| Mitf_Ex8 _M-form | gDNA | GCCTGTCTGGTTGAAGTACG | CCCTTATGGCTCATTACACC | 411 bp | 57 |
| Mitf_Ex9_1_M-form | gDNA | TTAAGTGTCTGATTTCTGTTTTCC | AAGGCAGTACCAATGAATCG | 983 bp | 57 |
| Mitf_Ex9_2_M-form | gDNA | AAGAGGATTTTGGCGTCAG | TTTGAAAGCCAGTTTTAGCC | 980 bp | 57 |
| Mitf_Ex9_3_M-form | gDNA | AGTGGTTTTCTGCATTCTTTG | TTTCCAAGGATTCTCATTGC | 948 bp | 57 |
| Mitf_Ex9_4_M-form | gDNA | GAATTGGTGATGGGTATTGG | AGGCAGGTGTACAAGTTTGG | 830 bp | 58 |
| Mitf_Ex9_4_M-form | gDNA | CTGTTTCTCCATCCCCTTC | TCTGAGTGCAAGTCATAAAGTAATC | 937 bp | 57 |
| cMITF_Ex1-3_iso-1 | cDNA | GGGCTCGGTTCTCAGTTTGG | CTCTCCGCCCTGTTTTGCTC | 541 bp | 62 |
| cMITF_Ex1-4_iso-2 | cDNA | GAGAGAGGGTGCATGTAAGC | ATCAAGCCCAGGATTTCTTC | 747 bp | 58 |
| cMITF_Ex2-4_iso-3 | cDNA | TATGACATCACGCATCTTGC | ATCAAGCCCAGGATTTCTTC | 579 bp | 58 |
| cMITF_Ex1-5_iso-7 | cDNA | AAAGATGGAGGCGCTTAGAG | GGCAGGAGTTACTGATGGTG | 750 bp | 58 |
| cMITF_Ex6-9 | cDNA | AGTCTGAAGCGAGAGCATTG | CTTTGGAGGCATTTGTAGTTG | 898 bp | 57 |
| MITF_SNP_629G>T | gDNA | GCACCATTACTAATAGCCCTTTCCT | GTACCTAGTTCCTTAATGCGGTCAT | 94 bp | 60 |
| MITF_Probe_G_Vic | gDNA | TGAACGAAGAA**G**AAGATTT |  |  |  |
| MITF_Probe_T_Fam | gDNA | TTGAACGAAGAA**T**AAGATTT |  |  |  |
