# Supplementary material for: A MITF Mutation Associated with a Dominant White Phenotype and Bilateral Deafness in German Fleckvieh Cattle
Source: PLoS One. 2011 Dec 12;6(12):e28857. doi: 10.1371/journal.pone.0028857 (PMC3236222; doi:10.1371/journal.pone.0028857)
Supplement: Table S3 — Number of animals (n) per breed, their coat color and genotype for the c.629G>T MITF mutation. (DOC) [file pone.0028857.s005.doc]

**Table S3.** Number of animals (n) per breed, their coat color and genotype for the c.629G>T *MITF* mutation.

| Breed | n | Phenotype | Genotype |
| --- | --- | --- | --- |
| German White Fleckvieh | 9 | white | G/T |
| German Fleckvieh related with German White Fleckvieh | 6 | spotted | G/G |
| German Fleckvieh unrelated with German White Fleckvieh | 89 | spotted | G/G |
| German Fleckvieh x Red Holstein | 7 | spotted | G/G |
| German Holstein | 40 | spotted | G/G |
| German Brown | 22 | brown | G/G |
| German Red Cattle | 26 | red | G/G |
| German Gelbvieh | 42 | yellow | G/G |
| Pinzgau | 3 | spotted | G/G |
| Blonde d’Aquitaine | 3 | blond | G/G |
| Charolais | 82 | creme-white | G/G |
| Limousin | 48 | blond | G/G |
| White Park | 15 | white-black markings | G/G |
| All non-completely white animals | 383 |  | G/G |
